# Sustainable Mite Management in Apple Orchards Under Climatic Stress: Ecological Trade-Offs and System Challenges

**DOI:** 10.3390/insects17070697

**Published:** 2026-07-04

**Authors:** Assel A. Karabayeva, Bakyt K. Kopzhassarov, Gulzhan B. Sarseyeva, Gulnar K. Ziyayeva, Assem D. Nogerbek, Aizhan K. Baubekova

**Affiliations:** 1 Department of Biology, M.Kh. Dulaty Taraz University, Taraz 080000, Kazakhstan; guljan-car@mail.ru (G.B.S.); gk.ziyaeva@dulaty.kz (G.K.Z.); ad.nogerbek@dulaty.kz (A.D.N.); 2 Zh. Zhiembayev Kazakh Research Institute of Plant Protection and Quarantine, Almaty 050070, Kazakhstan; bakyt-zr@mail.ru

**Keywords:** phytophagous mites, apple orchards, climate change, agroecosystem resilience, biological control, sustainable pest management

## Abstract

Climate change is increasingly altering the ecological dynamics of apple orchard ecosystems, creating new challenges for sustainable management of phytophagous mites. Rising temperatures, prolonged drought periods, and increasing climatic variability influence mite population dynamics, destabilize predator–prey interactions, and reduce the effectiveness of traditional pest management approaches. This review examines sustainable mite management in apple orchards through the interconnected perspectives of ecological stability, climatic stress, and resilience-oriented agroecosystem management. Particular attention is given to the ecological mechanisms underlying mite outbreaks, including climate-driven acceleration of reproduction, trophic destabilization, biodiversity loss, and disruption of biological regulation processes. The ecological limitations of both conventional chemical control and biological control strategies are critically analyzed, highlighting issues related to pesticide-induced ecological disturbance, resistance development, climatic sensitivity of natural enemies, and operational constraints. The review further explores resilience-oriented management frameworks based on ecological intensification, habitat diversification, conservation biological control, adaptive management, and system-oriented regulation. Current research gaps are identified, including the lack of long-term ecological studies, insufficient integration of climatic and ecological datasets, limited development of resilience indicators, and underrepresentation of continental and semi-arid orchard systems. The findings suggest that future sustainable mite management should move beyond reactive pest suppression toward ecosystem-based approaches that strengthen ecological resilience and adaptive capacity under increasing climatic uncertainty.

## Simple Summary

Apple orchards around the world are increasingly affected by climate change. Higher temperatures, drought, and changing weather patterns can promote the development of plant-feeding mites, which damage leaves, reduce tree productivity, and decrease fruit quality. Traditional control methods often rely on pesticides, but their repeated use may lead to resistance, harm beneficial organisms, and reduce the ecological stability of orchard ecosystems. This review examines how climate change influences mite outbreaks and discusses sustainable approaches to orchard protection. Special attention is given to the role of biodiversity, natural enemies, habitat management, and ecosystem-based practices that help improve the natural regulation of pest populations. The review also highlights current challenges and research gaps, including the need for long-term monitoring and climate-adaptive management strategies. Understanding these ecological processes can help develop more sustainable and resilient orchard systems, reduce dependence on pesticides, and support stable fruit production under changing environmental conditions.

## 1. Introduction

Apple orchards represent one of the most economically important perennial agroecosystems worldwide, yet their sustainability is increasingly threatened by climate-related stressors and destabilization of pest regulation processes. Among the most persistent arthropod pests, phytophagous mites, particularly the European red mite (*Panonychus ulmi*) and spider mites (*Tetranychus* spp.), continue to cause substantial reductions in fruit quality, photosynthetic activity, and orchard productivity in temperate fruit-growing regions. Recent studies indicate that mite outbreaks are becoming more frequent and less predictable under conditions of climatic variability, especially in intensive orchard systems characterized by simplified vegetation structure and high anthropogenic pressure [1,2].

Conventional mite management in apple orchards has historically relied on repeated acaricide applications due to their rapid suppressive effect and operational convenience. However, increasing evidence suggests that intensive chemical control may negatively affect non-target organisms, contribute to resistance development, and weaken long-term ecological regulation processes within orchard ecosystems [3,4]. These concerns have stimulated growing interest in more sustainable and ecologically compatible management approaches.

In response to these limitations, sustainable orchard protection increasingly emphasizes ecological regulation and conservation biological control. Predatory mites of the family *Phytoseiidae* are recognized as key natural enemies of phytophagous mites in perennial crops. Nevertheless, the effectiveness of biological control varies considerably among orchard systems and depends on ecological and management conditions, highlighting the need for a broader ecosystem-based perspective on mite regulation [5].

Climate change further complicates sustainable mite management by affecting both pest populations and the ecological processes responsible for their regulation [6]. Rising temperatures, drought, and increasing climatic variability may alter species interactions and reduce the stability of orchard ecosystems, particularly in continental and semi-arid fruit-growing regions [7,8,9,10]. Under these conditions, mite management becomes not only a pest-control issue but also a challenge of maintaining ecological resilience.

Although sustainable orchard management has received increasing attention, existing reviews have primarily focused on integrated pest management techniques or specific biological control agents. Less attention has been paid to the broader ecological mechanisms linking climate change, trophic stability, biodiversity, and long-term resilience of orchard ecosystems [11,12,13].

Therefore, this review examines sustainable mite management in apple orchards through the interconnected perspectives of ecological trade-offs, climatic stress, and system-level challenges. Rather than providing a conventional overview of control methods, the article focuses on the ecological mechanisms that destabilize mite regulation in orchard systems and discusses how resilience-oriented approaches may contribute to long-term sustainability under increasing climatic uncertainty.

## 2. Ecological Stability and Trade-Offs in Orchard Systems

The ecological sustainability of fruit agroecosystems in modern conditions is considered a key condition for the long-term effectiveness of horticulture. Unlike short-term intensive production models based primarily on chemical pest control, resilient orchard systems are characterized by the ability to maintain functional ecological relationships even when exposed to external stress factors. This issue is particularly significant for perennial apple plantations, as ecosystem stability develops over a long period of time and is determined by a complex set of interactions between plants, phytophages, predators, soil biota, and the structure of the agricultural landscape. Disruption of these interactions can lead to a sharp increase in pest populations, including phytophagous mites, which are highly adaptable to changing environmental conditions.

Modern research increasingly demonstrates that the ecological sustainability of fruit orchards is closely associated with the level of functional biodiversity and the complexity of trophic interactions within agroecosystems [14,15]. Functional biodiversity is important not only because of the abundance of beneficial organisms but also due to its role in maintaining ecological regulation processes, including biological control, trophic buffering, and ecosystem resilience. In ecologically stable orchard systems, pest suppression is supported by the continuous presence of natural enemies, spatial heterogeneity of habitats, alternative food resources, and more stable microclimatic conditions. By contrast, simplified agricultural systems characterized by intensive anthropogenic intervention often exhibit weakened self-regulation capacity and increased vulnerability to pest outbreaks [16].

Recent ecological studies further suggest that agroecosystem resilience depends not only on species richness itself, but also on functional redundancy, whereby similar ecological functions are maintained by multiple groups of organisms simultaneously [17]. Such redundancy enhances the ability of agroecosystems to preserve biological regulation under environmental disturbances and partial disruption of trophic interactions. In intensive orchard systems, however, reductions in functional diversity and habitat complexity frequently increase ecosystem sensitivity to climatic variability and anthropogenic stressors. Functional simplification may therefore destabilize predator–prey interactions and reduce the ecological buffering capacity of orchard ecosystems [18].

Isbell et al. demonstrated that biodiversity significantly enhances the resistance of ecosystem productivity to climatic extremes, including drought and temperature anomalies [19]. The authors emphasized that biodiversity loss weakens ecological buffering mechanisms and decreases ecosystem capacity to withstand abrupt environmental disturbances. For perennial orchard systems, these processes may contribute to increased instability of phytophagous mite population dynamics under changing climatic conditions. Similar conclusions were reported by Allan et al., who showed that agricultural intensification alters ecosystem multifunctionality through biodiversity loss and shifts in functional composition, thereby reducing long-term ecological stability [20].

An important characteristic of perennial orchard ecosystems is the relatively long persistence of trophic interactions compared with annual cropping systems. Apple orchards may maintain populations of predators, parasitoids, and soil organisms over multiple vegetation seasons, which potentially increases the stability of biological regulation processes. However, such long-term ecological continuity also means that disturbances associated with excessive pesticide use, vegetation removal, or climatic stress can accumulate over time and gradually destabilize ecosystem functioning. Dib et al. demonstrated that increased habitat heterogeneity contributed to greater trophic stability and resilience of ecological regulation processes in apple orchards [21]. The authors further noted that diversified orchard systems exhibited greater resilience to ecological disturbances than conventionally managed orchards with simplified vegetation structure.

The ecological significance of habitat diversification lies in its ability to support more complex trophic networks and provide refuges for beneficial organisms within orchard ecosystems. Increased ecological complexity may enhance the persistence of natural enemies, improve trophic buffering, and reduce the vulnerability of biological regulation processes to environmental disturbances. However, the ecological outcomes of diversification remain strongly context-dependent and are influenced by landscape structure, climatic conditions, and management intensity [22].

One of the major ecological trade-offs in modern horticulture arises from the conflict between short-term productivity optimization and long-term ecosystem resilience. Intensive orchard systems are typically designed to maximize production efficiency through dense planting schemes, intensive irrigation, mechanized management, and frequent pesticide applications. Although these approaches may increase productivity in the short term, they often alter orchard microclimate, simplify trophic networks, and reduce ecological buffering capacity. High-density orchards with limited understory vegetation are particularly vulnerable to rapid pest population growth because of increased canopy temperatures and reduced habitat availability for natural enemies [13]. Such conditions may increase ecosystem vulnerability to pest outbreaks while simultaneously weakening biological regulation processes.

In recent years, increasing attention has been paid to the role of ecosystem multifunctionality in agricultural sustainability. Modern agroecological concepts suggest that sustainable orchard systems should not be evaluated solely by yield performance, but also by their ability to simultaneously maintain biodiversity, pest regulation, soil quality, and resilience to climatic stress. Manning et al. emphasized that ecosystem multifunctionality depends strongly on biodiversity and functional interactions among organisms, and that intensification-driven biodiversity loss may compromise multiple ecosystem services simultaneously [23]. In orchard agroecosystems, this implies that excessive simplification aimed at maximizing production may ultimately reduce long-term ecological and economic sustainability.

Additional complexity emerges from interactions between climate change and agricultural intensification. Climatic warming, altered precipitation regimes, and increasing frequency of extreme weather events may amplify existing ecological instabilities in orchard systems. Because different components of agroecosystems respond unequally to climatic stress, environmental change may alter trophic relationships and reduce ecosystem stability. These interactions are particularly important in continental and semi-arid fruit-growing regions, where climatic extremes increasingly interact with intensive management practices [24].

**Figure 1:**
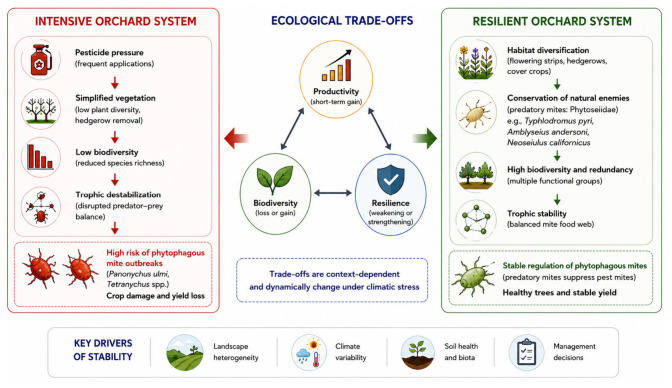
Ecological stability and trade-offs in apple orchard systems.

The interactions among biodiversity, management intensity, and ecological resilience in orchard systems are summarized in Figure 1.

Figure 1 illustrates that ecological stability in orchard systems depends on the balance between management intensity, biodiversity conservation, and resilience-oriented regulation processes. Intensive orchard management may increase short-term productivity but simultaneously reduce trophic stability and ecological buffering capacity. In contrast, diversified orchard systems support functional biodiversity and biological regulation, thereby enhancing long-term ecosystem resilience under climatic stress. However, the effectiveness of such systems remains strongly dependent on landscape context, climatic conditions, and management practices.

Overall, ecological stability in apple orchards depends on complex interactions among biodiversity, habitat structure, climatic variability, and management intensity. Phytophagous mite outbreaks should therefore be interpreted not only as isolated pest problems, but also as indicators of broader ecosystem destabilization. These ecological interactions become particularly vulnerable under climatic stress, where warming and drought may further amplify trophic instability within orchard agroecosystems.

## 3. Sustainable Mite Management Under Climatic Stress

### 3.1. Climate-Driven Changes in Mite Population Dynamics

Climate change is increasingly recognized as one of the major drivers influencing the population dynamics of phytophagous mites in orchard ecosystems. Rising temperatures, altered precipitation regimes, prolonged drought periods, and increasing frequency of extreme weather events significantly modify the interactions among host plants, phytophagous mites, and their natural enemies. In perennial orchard systems, these changes may destabilize long-established trophic relationships and contribute to the emergence of more frequent and severe mite outbreaks. Unlike many insect pests, phytophagous mites possess short generation times, rapid reproductive capacity, and high physiological plasticity, which allow them to respond quickly to environmental fluctuations and exploit favorable climatic conditions.

One of the most important consequences of climatic warming is the acceleration of mite developmental rates. Temperature directly affects embryonic development, larval survival, fecundity, dispersal capacity, and generation turnover of phytophagous mites. Under elevated temperatures, developmental periods become shorter, allowing multiple overlapping generations during the growing season and increasing the probability of rapid population growth. Recent studies have shown that warming conditions may significantly increase the reproductive performance of *Tetranychus urticae* and related phytophagous mite species, especially under dry environmental conditions. Migeon et al. demonstrated that mites feeding on drought-stressed plants developed faster and exhibited increased fecundity compared with individuals feeding on well-watered plants [8]. The authors further noted that climatic origin influenced the adaptive responses of mite populations, suggesting that populations from warmer and drier regions may possess greater tolerance to climatic stress.

In orchard ecosystems, climatic warming additionally modifies seasonal phenology and may contribute to earlier onset of mite infestations. Higher spring temperatures can accelerate the emergence and colonization of leaves by phytophagous mites, extending the duration of feeding activity during the vegetation period. Such shifts increase cumulative plant damage and intensify pressure on orchard ecosystems. Moreover, elevated temperatures frequently enhance the suitability of orchard microclimates for mite development, particularly in intensive plantations characterized by reduced canopy ventilation and simplified understory vegetation. These conditions create favorable thermal environments for phytophagous mites while simultaneously reducing ecological buffering capacity. Importantly, climate-driven acceleration of mite development may destabilize traditional timing-based management strategies in orchard systems. Under warming conditions, rapid generation turnover may increase the frequency and intensity of mite outbreaks, making population growth less predictable and reducing the effectiveness of conventional timing-based management strategies. These changes increase the unpredictability of mite outbreaks and complicate population management in orchard systems.

Drought stress represents another major climatic factor affecting mite population dynamics in perennial crops. Water-deficient plants often undergo physiological and biochemical changes that alter plant–herbivore interactions. Reduced water availability may decrease plant resistance mechanisms, modify nutrient composition, and increase the concentration of soluble nitrogen compounds in plant tissues, thereby improving host suitability for phytophagous mites. In addition, drought conditions are commonly associated with reduced relative humidity, which may further promote mite development and survival. These findings indicate that drought should not be considered solely as an abiotic stress affecting plant physiology. In orchard ecosystems, drought simultaneously alters plant nutritional quality, microclimatic conditions, and predator–prey interactions, thereby functioning as a multidimensional ecological destabilization factor. Consequently, drought-associated mite outbreaks may reflect broader ecosystem vulnerability rather than isolated pest responses alone.

Barnes et al. reported that drought stress substantially increased the susceptibility of orchard systems to spider mite outbreaks by altering host plant physiology and enhancing environmental suitability for mite reproduction [25]. Similar observations were reported in almond orchards, where severe drought years were associated with dramatic increases in the population density of *Tetranychus urticae* and other phytophagous mites. Saeidi et al. demonstrated that under drought conditions and elevated temperatures, spider mite populations increased several-fold compared with years characterized by normal precipitation patterns [26]. The authors also noted that drought-stressed orchards exhibited earlier seasonal infestation and accelerated population growth, indicating strong climatic control over mite dynamics.

Another important aspect of climate-driven mite outbreaks involves the interaction between climatic stress and agricultural intensification. Intensive orchard systems often possess simplified vegetation structure, reduced habitat heterogeneity, and elevated canopy temperatures, which together amplify the ecological effects of climatic warming. Dust accumulation, water stress, and reduced biodiversity may further increase the vulnerability of such systems to phytophagous mite outbreaks. Studies conducted in apple orchards of dry inland regions showed that dust deposition and drought conditions significantly increased phytophagous mite abundance while negatively affecting natural enemies [24]. These findings indicate that climatic and anthropogenic stressors frequently act synergistically rather than independently.

Climate change may also contribute to the geographic expansion of mite pests into regions previously considered climatically unsuitable. Increasing temperatures facilitate the establishment of thermophilic species in temperate fruit-growing areas and may alter the distribution patterns of economically important phytophagous mites. Recent studies suggest that climatic warming and increasing environmental stress may facilitate the expansion and establishment of economically important phytophagous mite species in previously less favorable agricultural regions [27]. Such processes may increase future risks for perennial orchard systems, particularly in continental and semi-arid fruit-growing regions already experiencing climatic instability.

Recent studies from Asia further support the importance of climate-driven shifts in mite population dynamics [28,29]. Chen et al. demonstrated that temperature and precipitation are key determinants of the future distribution of the predatory mite *Neoseiulus californicus* in China, suggesting that climate change may substantially alter biological regulation processes in orchard ecosystems. Similar concerns have been raised for East Asian fruit-growing regions, where changing climatic conditions are expected to influence both pest and natural enemy distributions [30].

Overall, climatic stress substantially alters the population dynamics of phytophagous mites by accelerating reproduction, shortening developmental cycles, weakening host plant resistance, and destabilizing biological regulation processes. Under conditions of ongoing climate change, sustainable mite management in orchard systems increasingly depends on understanding the ecological interactions between climatic variables, agroecosystem structure, and trophic regulation. These processes suggest that future orchard protection strategies should move beyond reactive pest suppression toward adaptive and climate-resilient management approaches capable of maintaining ecological stability under rapidly changing environmental conditions.

**Figure 2:**
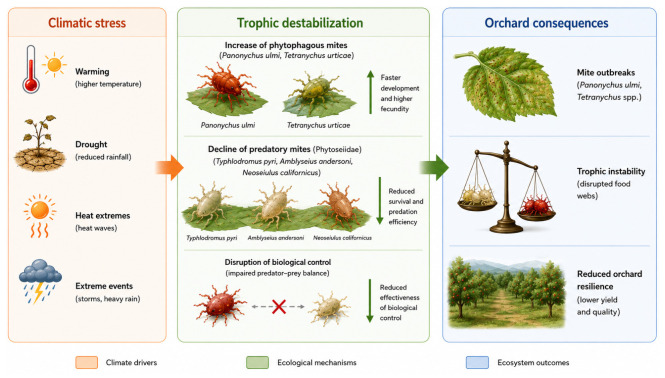
Effects of climatic stress on trophic destabilization and ecological resilience in orchard mite communities.

The climate-driven mechanisms underlying trophic destabilization and ecological vulnerability in orchard mite communities are summarized in Figure 2.

### 3.2. Climatic Effects on Biological Regulation

While Section 3.1 focused on the direct effects of climatic stress on phytophagous mite populations, climate change also affects the ecological mechanisms responsible for their regulation. In orchard ecosystems, biological control depends on stable predator–prey interactions, yet increasing climatic variability may alter the survival, phenology, and effectiveness of natural enemies, thereby destabilizing trophic regulation processes [31].

Experimental evidence indicates that climate warming can modify intraguild interactions among predatory mites. Urbaneja-Bernat et al. demonstrated that future warming scenarios influenced survival, oviposition, and competitive interactions among phytoseiid species involved in the regulation of *Tetranychus urticae* populations [32]. The authors concluded that climate-driven abiotic changes may modify the structure of predatory mite communities and potentially destabilize biological control outcomes in orchard systems. Similarly, Liao et al. emphasized that changing temperature regimes and extreme climatic events may significantly reduce the biological control potential of predatory mites, thereby creating additional challenges for sustainable pest management under climate change [33]. Taken together, these studies indicate that climate change may reduce the reliability of biological control in perennial orchard systems. Notably, ecological destabilization does not necessarily occur through complete collapse of predator populations, but rather through disruption of synchronization among trophic levels. Even moderate shifts in temperature or humidity may alter reproductive timing, dispersal behavior, and competitive interactions among predatory mites, potentially leading to reduced regulation efficiency despite continued predator presence.

As a result, climatic change increasingly transforms biological regulation from a relatively stable ecological process into a highly variable and environmentally dependent mechanism. One of the major ecological consequences of climatic warming is the disruption of synchrony between phytophagous mites and their predators. Biological control in orchard ecosystems depends strongly on temporal coordination between prey availability and predator activity. Elevated temperatures may alter the temporal synchrony between predators and prey, thereby reducing the efficiency of biological regulation during critical periods of orchard development [34]. Under such conditions, predators may fail to suppress rapidly expanding phytophagous populations during critical periods of orchard development.

Sentis et al. demonstrated that warming conditions may substantially alter trophic interaction strength and destabilize predator–prey regulation within arthropod communities [9]. The authors reported that elevated temperatures increased herbivore performance while simultaneously modifying predation efficiency and competitive interactions among predators. These findings suggest that climatic warming may not only increase pest abundance directly, but also indirectly weaken ecological regulation mechanisms that normally stabilize orchard systems. Additional complexity arises from the differential sensitivity of predatory mites and phytophagous mites to humidity and temperature fluctuations. Many phytoseiid mites require relatively stable humidity conditions for optimal survival and reproduction, whereas several phytophagous species demonstrate greater tolerance to drought and thermal stress [35]. Consequently, prolonged heat waves and reduced air humidity may create ecological conditions favoring pest development over predator persistence. This imbalance becomes especially problematic in intensive orchard systems characterized by simplified vegetation structure and reduced microclimatic buffering. Environmental stress may substantially reduce the effectiveness of predatory mites used in biological control programs. Ghazy et al. reviewed evidence showing that environmental stressors significantly affect the biological performance of phytoseiid mites and may compromise the long-term stability of biological control systems under unfavorable environmental conditions [35]. Even where predatory mites are initially abundant, climatic stress may reduce their establishment success and long-term persistence.

An additional problem associated with climatic change involves the increasing frequency of extreme weather events. Short-term heat extremes may produce rapid ecological disturbances within orchard ecosystems by affecting mortality, dispersal behavior, and reproductive performance of beneficial arthropods [36]. Unlike gradual climatic warming, episodic climatic extremes often generate abrupt ecological responses that are difficult to predict using conventional pest management models. Recent ecological studies suggest that extreme temperature events may disproportionately affect higher trophic levels, including predators and parasitoids, thereby reducing the resilience of biological regulation systems [37].

Ma et al. reported that short-term thermal extremes negatively influenced survival and predatory efficiency of natural enemies more strongly than those of herbivorous arthropods [38]. Such asymmetric responses may create ecological windows during which phytophagous mite populations rapidly escape biological regulation. In perennial orchard systems, repeated exposure to climatic extremes may therefore contribute to chronic destabilization of trophic interactions and increasing unpredictability of pest outbreaks. Importantly, climatic effects on biological regulation are strongly influenced by orchard structure and landscape context. Ecologically diversified orchards containing ground vegetation, flowering strips, and semi-natural habitats often maintain more stable microclimatic conditions than highly simplified orchard systems. Vegetation complexity may reduce thermal fluctuations, increase air humidity, and provide refuges for predatory arthropods during periods of climatic stress [39]. In contrast, orchards characterized by bare soil management and intensive canopy simplification frequently exhibit elevated temperatures and reduced ecological buffering capacity.

Recent landscape-scale studies indicate that heterogeneous agricultural landscapes may partially mitigate the destabilizing effects of climate change on biological control [40]. Muneret et al. demonstrated that landscape complexity enhanced the abundance and stability of natural enemy communities in perennial agricultural systems despite increasing environmental variability [41]. The authors emphasized that semi-natural habitats may function as climatic refugia that support predator persistence under stressful environmental conditions. These findings highlight the importance of integrating landscape ecology into sustainable orchard management strategies under climate change.

Continental orchard systems are considered particularly vulnerable to climate-driven destabilization of biological regulation [42]. Such systems are commonly characterized by large seasonal and daily temperature fluctuations, periodic drought stress, and relatively low atmospheric humidity. Under these conditions, ecological regulation processes may become increasingly unstable, especially in intensively managed orchards lacking sufficient habitat heterogeneity [43]. In Central Asian and other continental fruit-growing regions, climatic warming is expected to intensify water deficits and increase thermal stress during the vegetation season, potentially amplifying the ecological advantages of phytophagous mites over their natural enemies [44].

Another important issue concerns the adaptability of biological control strategies under rapidly changing climatic conditions. Many existing conservation biological control programs were developed under relatively stable climatic assumptions and may therefore become less reliable under future environmental scenarios. Climate change may alter not only predator efficiency, but also dispersal patterns, overwintering success, and interspecific interactions among beneficial arthropods [45]. Consequently, biological regulation in orchard ecosystems should increasingly be viewed as a dynamic and climate-sensitive process rather than a stable ecological service.

Recent studies further suggest that climate-driven trophic destabilization may reduce the predictability of integrated pest management programs [46]. Under variable climatic conditions, thresholds traditionally used for mite control decisions may no longer accurately reflect future outbreak risks because predator–prey relationships themselves become unstable [47]. This creates substantial challenges for sustainable orchard protection, particularly in regions already exposed to increasing climatic variability.

Overall, climatic stress significantly affects biological regulation in orchard ecosystems through disruption of predator–prey synchronization, reduction in predatory efficiency, and destabilization of trophic interactions. These effects are particularly pronounced in continental orchard systems exposed to high thermal variability and drought stress. Under ongoing climate change, sustainable mite management increasingly depends on the ability of orchard systems to maintain ecological buffering and trophic resilience despite intensifying environmental disturbances. Consequently, future orchard protection strategies should focus not only on direct suppression of phytophagous mites, but also on preserving ecological conditions that sustain long-term biological regulation under climatic uncertainty.

## 4. Limitations and Challenges of Sustainable Mite Management

The increasing ecological instability of orchard agroecosystems has intensified discussions regarding the long-term sustainability of both conventional chemical control and biological regulation strategies. Although pesticide-based suppression remains one of the most widely applied approaches for managing phytophagous mites in commercial orchards, growing evidence indicates that intensive chemical interventions may simultaneously weaken the ecological mechanisms necessary for stable long-term pest regulation. At the same time, biological control systems, despite their ecological advantages, also demonstrate substantial limitations associated with climatic sensitivity, operational complexity, and inconsistent effectiveness under highly variable environmental conditions. Consequently, sustainable mite management increasingly requires critical reassessment of both conventional and biological control paradigms within the broader context of agroecosystem resilience.

### 4.1. Ecological Limitations of Chemical Control

One of the most significant ecological limitations of conventional mite management involves the disruption of trophic interactions caused by repeated pesticide applications [48]. Broad-spectrum acaricides frequently affect not only target phytophagous mites but also predatory mites, parasitoids, pollinators, and other non-target arthropods involved in ecological regulation processes. Such disturbances may destabilize orchard food webs and reduce the natural suppression capacity of agroecosystems. The ecological consequences of pesticide disturbance are particularly important in perennial orchard systems, where long-term biological interactions develop gradually over multiple vegetation seasons.

Recent studies have demonstrated that pesticide applications may significantly alter the abundance and structure of beneficial arthropod communities in orchards [49,50]. In apple orchards, reductions in phytoseiid mite populations following pesticide applications have frequently been associated with weakened biological regulation and increased risks of secondary outbreaks of *Panonychus ulmi* and *Tetranychus* spp. Such effects highlight the importance of preserving predatory mite communities as a key component of sustainable orchard protection.

Biondi et al. emphasized that many insecticides and acaricides negatively affect non-target natural enemies even at sublethal concentrations, influencing survival, fecundity, dispersal behavior, and predatory activity [51]. Notably, sublethal effects often remain less visible than direct mortality but may substantially reduce long-term biological regulation efficiency. In orchard ecosystems, repeated exposure to chemical stress may therefore progressively weaken ecological buffering mechanisms despite short-term suppression of pest populations.

Another major limitation associated with intensive acaricide use is the rapid development of resistance in phytophagous mite populations [52]. Species such as *Tetranychus urticae* and *Panonychus ulmi* possess high reproductive potential, short generation cycles, and considerable genetic plasticity, making them particularly prone to rapid adaptation under pesticide pressure. Resistance development has become one of the most serious challenges in modern orchard protection, especially in intensive production systems characterized by repeated applications of chemically similar compounds [53].

Van Leeuwen et al. demonstrated that *Tetranychus urticae* exhibits exceptional evolutionary adaptability to acaricides because of complex detoxification mechanisms, target-site mutations, and metabolic resistance pathways [54]. The authors further noted that resistance evolution frequently outpaces the development of new acaricidal compounds, thereby reducing the long-term effectiveness of chemical control programs. Similar conclusions were reported by Dermauw et al., who emphasized that resistance mechanisms in spider mites increasingly involve multiple interacting physiological pathways rather than isolated genetic mutations [55]. Such multidimensional resistance substantially complicates sustainable mite management and often necessitates increasing pesticide application frequency or dosage, thereby intensifying ecological disturbance within orchard systems.

Importantly, pesticide-driven ecological destabilization may itself contribute indirectly to secondary mite outbreaks. Elimination of predatory mites and disruption of trophic regulation may create ecological conditions favorable for rapid recovery of phytophagous mite populations following chemical treatments [56]. This phenomenon has been documented in multiple orchard systems where pesticide applications initially reduced pest abundance but subsequently triggered resurgence effects associated with weakened biological regulation.

Hardman et al. reported that pesticide disturbance significantly altered predator–prey relationships in apple orchards and contributed to secondary outbreaks of *Panonychus ulmi* under certain management regimes [57]. The authors emphasized that the ecological consequences of pesticide applications frequently extend beyond immediate toxicological effects and may involve long-term destabilization of orchard trophic interactions. These findings suggest that intensive chemical suppression may paradoxically increase future dependence on pesticide applications by weakening natural ecological regulation processes.

### 4.2. Ecological and Operational Limitations of Biological Control

Biological control strategies are often presented as environmentally safer alternatives to conventional pesticide-based management. Predatory mites, conservation biological control, habitat diversification, and augmentation programs may reduce pesticide dependence and support ecological resilience in orchard systems [12]. However, biological regulation also demonstrates considerable limitations that are sometimes underestimated in sustainable agriculture discourse. In many orchard systems, biological control effectiveness remains highly variable and strongly dependent on climatic conditions, habitat structure, management intensity, and synchronization between predators and prey [58]. In apple orchards, predatory mites such as *Typhlodromus pyri*, *Amblyseius andersoni*, and *Neoseiulus californicus* are among the most important natural enemies of phytophagous mites and constitute a central component of conservation biological control programs. However, the effectiveness of these species may vary considerably depending on climatic conditions, pesticide compatibility, and habitat availability.

One of the primary operational limitations of biological control involves the instability of predator establishment under fluctuating environmental conditions. Successful regulation by predatory mites often requires relatively stable humidity, suitable overwintering habitats, adequate prey availability, and minimal pesticide disturbance. Under intensive orchard conditions, these ecological requirements are frequently not fully maintained. Consequently, biological control programs may demonstrate inconsistent effectiveness among years, regions, and orchard systems. Knapp et al. emphasized that the establishment and long-term persistence of phytoseiid mites are strongly influenced by environmental conditions and orchard management practices, which may substantially affect biological regulation efficiency under commercial production systems [59]. Importantly, the ecological effectiveness of biological regulation does not always correspond to operational predictability required in intensive horticulture. While conservation biological control may improve long-term ecosystem resilience, its short-term outcomes are often less immediate and less consistent than those of chemical suppression. This creates a practical conflict between ecological sustainability and production reliability, particularly in highly intensive orchard systems exposed to climatic variability and economic pressure.

Duso et al. emphasized that the effectiveness of predatory mites in orchards is strongly influenced by environmental heterogeneity, pesticide compatibility, and microclimatic conditions [60]. The authors noted that biological regulation in perennial crops should not be interpreted as a universally stable process, since ecological performance of predatory mites may vary substantially under different management systems. This variability creates practical difficulties for commercial orchard production, where growers often require rapid and predictable suppression of economically important pests.

An additional ecological concern involves the unintended consequences associated with certain biological control introductions. Although augmentation and release of predatory mites may improve suppression of phytophagous pests, introduced biological control agents can occasionally interfere with existing trophic interactions or compete with indigenous natural enemies [61]. Such ecological effects remain insufficiently investigated in many orchard systems, particularly under conditions of climatic instability and increasing environmental stress. In some cases, introduced predators may alter community structure, modify intraguild interactions, or displace native predatory species, thereby generating ecological outcomes that are difficult to predict under field conditions [62]. Importantly, climate change may further increase uncertainty associated with introduced biological control agents. Altered temperature regimes, changing species distributions, and modified trophic interactions may influence establishment success and ecological behavior of introduced predators in ways that remain poorly understood. Consequently, future sustainable mite management strategies should incorporate not only pest suppression efficacy but also long-term ecological risk assessment and post-release ecosystem monitoring.

Roy et al. highlighted that biological control programs may produce unintended ecological effects when introduced natural enemies interact with native arthropod communities in unpredictable ways [63]. While most classical biological control programs remain beneficial overall, the authors emphasized the importance of long-term ecological assessment and post-release monitoring. In orchard ecosystems characterized by climatic instability and increasing environmental stress, such ecological uncertainties may become more pronounced.

### 4.3. Socio-Economic and Implementation Barriers

Economic and operational constraints further complicate large-scale implementation of biological control strategies. Conservation biological control and habitat diversification frequently require long-term management adjustments, additional monitoring, specialized expertise, and reduced reliance on conventional pesticide programs. Although such approaches may improve ecological sustainability, short-term economic benefits are not always immediately evident to producers. In highly competitive fruit markets, growers may therefore prioritize immediate production stability over longer-term ecological resilience.

Recent analyses indicate that implementation barriers remain one of the major obstacles limiting widespread adoption of ecological pest management in commercial horticulture. Lamichhane et al. emphasized that integrated and biological approaches often face constraints related to economic uncertainty, knowledge transfer, regulatory limitations, and inconsistent field performance [64]. The authors argued that sustainable pest management should not be viewed solely as a technological problem, but also as a socio-economic and management challenge requiring systemic transformation of agricultural practices. Similar conclusions were reported by Pretty and Bharucha, who noted that ecological intensification strategies often require systemic changes in farm management, farmer training, and institutional support before long-term economic and ecological benefits become fully realized [65]. These findings suggest that sustainable pest management should not be viewed solely as a technological issue, but rather as a broader socio-economic and management challenge requiring transformation of production systems, decision-making frameworks, and agricultural policy.

Another important limitation concerns the conceptual overreliance on single-method solutions within sustainable agriculture discourse. Neither chemical suppression nor biological control alone appears capable of ensuring stable long-term regulation of phytophagous mites under rapidly changing climatic conditions [66,67]. Conventional pesticide-based management often accelerates ecological simplification and resistance development, whereas biological control systems may become increasingly unstable under climatic stress and intensive production conditions. Consequently, sustainable mite management requires integrated and adaptive approaches capable of balancing ecological resilience with operational reliability.

The major ecological trade-offs associated with conventional and biological mite management strategies are summarized in Table 1.

Recent ecological studies increasingly support the view that orchard sustainability depends not on complete replacement of chemical control, but on reducing ecological disruption while maintaining trophic regulation capacity within agroecosystems [68,69]. Under this perspective, the principal challenge is not simply suppression of phytophagous mites, but maintenance of ecological stability despite climatic variability, resistance evolution, and production intensification. Contemporary agroecological frameworks increasingly emphasize that sustainable pest management should enhance ecosystem resilience, biodiversity-mediated regulation, and adaptive capacity rather than rely exclusively on reactive pest eradication strategies [70]. This conceptual shift fundamentally transforms orchard protection from a chemically centered suppression model toward resilience-oriented ecosystem management.

## 5. Toward Resilience-Oriented Orchard Management

The increasing ecological instability of orchard agroecosystems under climatic change has stimulated a gradual transition from conventional pest suppression models toward resilience-oriented management frameworks. In contrast to traditional protection strategies primarily focused on short-term eradication of economically important pests, resilience-oriented orchard management emphasizes the capacity of agroecosystems to maintain ecological functions, trophic regulation, and adaptive stability under variable environmental conditions. Within this perspective, sustainable mite management is no longer interpreted solely as a technical issue of population suppression, but rather as a broader ecological challenge involving maintenance of biodiversity, ecosystem services, and long-term agroecosystem functionality.

The concept of ecological intensification has become a central component of contemporary agroecological research. Ecological intensification does not aim to eliminate agricultural productivity goals, but rather seeks to enhance ecosystem services that contribute to long-term system stability while reducing ecological disruption associated with intensive external inputs. In orchard ecosystems, ecological intensification may involve strengthening biological regulation, improving habitat heterogeneity, conserving beneficial arthropods, and increasing trophic complexity within the agricultural landscape.

Tittonell emphasized that ecological intensification should be understood as a process of redesigning agroecosystems to improve ecological functionality rather than simply substituting one management input for another [70]. According to this framework, sustainable agricultural systems depend on strengthening adaptive ecological processes capable of buffering environmental stress and maintaining system resilience under changing climatic conditions. Similarly, Wezel et al. noted that agroecological intensification increasingly relies on ecosystem-based regulation processes, including biological control, habitat diversification, nutrient cycling, and biodiversity-mediated stability [71]. These studies collectively suggest that sustainable orchard protection requires a transition from chemically centered management toward ecologically integrated regulation systems.

One of the most important components of resilience-oriented orchard management involves habitat diversification. Simplified orchard systems characterized by bare soil management, homogeneous vegetation structure, and intensive pesticide use frequently exhibit reduced trophic complexity and weakened ecological buffering capacity. By contrast, diversified orchard systems containing flowering strips, ground cover vegetation, hedgerows, and semi-natural habitats may support greater abundance and stability of beneficial arthropods, including predatory mites and insect natural enemies.

Recent studies demonstrate that habitat diversification may improve the persistence of biological regulation processes under environmental stress. Tschumi et al. reported that ecological compensation areas and diversified habitats enhanced natural enemy abundance and improved biological pest suppression in agricultural landscapes [72]. Importantly, the authors emphasized that diversified habitats function not only as reservoirs of beneficial organisms, but also as ecological stabilizers that reduce vulnerability of agroecosystems to environmental disturbances. Similar conclusions were presented by Landis, who argued that habitat management contributes to resilience by supporting trophic redundancy and reducing dependence on external chemical inputs [73].

Evidence from Australian apple orchards further supports the role of habitat diversification in strengthening biological regulation. Managing floral resources through flowering strips, hedgerows, and other habitat enhancements has been shown to support predatory arthropods and improve ecosystem services associated with pest suppression in orchard systems [74].

However, the ecological benefits of habitat diversification should not be interpreted as universally predictable or uniformly effective across orchard systems. The effectiveness of diversification strategies may vary among orchard systems according to climatic and landscape conditions. In some cases, increased vegetation complexity may simultaneously support beneficial arthropods and alternative pest species, thereby generating ecological trade-offs that complicate management outcomes.

Ecological responses to diversification strategies vary substantially among orchard systems. Karp et al. demonstrated that ecological responses to diversification strategies vary substantially among agricultural systems and may depend on interactions between local habitat management and surrounding landscape composition [75]. The authors emphasized that successful ecological intensification requires adaptive integration of ecological principles with region-specific production conditions rather than universal application of standardized biodiversity measures. These findings are particularly important for continental orchard systems, where climatic variability may substantially alter the effectiveness of ecological regulation processes among years.

Conservation biological control has become another central component of resilience-oriented orchard management. Unlike augmentation programs focused primarily on periodic release of predatory organisms, conservation biological control aims to maintain and enhance naturally occurring populations of beneficial arthropods through ecological management of habitats and reduction in disturbances. In orchard systems, this approach frequently includes minimizing pesticide pressure, preserving overwintering habitats, maintaining alternative food resources, and improving structural heterogeneity within agroecosystems.

Several studies have highlighted the ecological advantages of conservation-based regulation strategies. Deguine et al. emphasized that conservation biological control contributes not only to pest suppression, but also to broader ecosystem resilience by stabilizing trophic interactions and enhancing adaptive ecological capacity [76]. The authors argued that long-term sustainability of agricultural systems depends largely on maintaining functional ecological communities capable of responding dynamically to environmental disturbances. Under climate change, such adaptive ecological capacity may become increasingly important for reducing vulnerability of orchard systems to pest outbreaks.

Nevertheless, conservation biological control also faces substantial practical and ecological limitations. Ecological regulation frequently develops gradually and may require long-term management continuity before measurable benefits become evident. In highly intensive commercial orchards, producers often prioritize immediate economic stability and predictable pest suppression, whereas ecological regulation processes may exhibit delayed or variable responses. Consequently, implementation of conservation biological control frequently depends on balancing ecological sustainability goals with operational and economic realities of fruit production systems.

### 5.1. Climate-Adaptive Monitoring of Phytophagous Mites

Effective resilience-oriented management of phytophagous mites requires climate-adaptive monitoring systems capable of detecting population changes before economically damaging outbreaks occur. Under climatic warming, traditional monitoring schedules based on historical phenological patterns may become increasingly unreliable because temperature affects developmental rates, generation turnover, and seasonal population dynamics of *Panonychus ulmi* and *Tetranychus* spp.

Regular monitoring of mite populations, assessment of predator–prey ratios, and integration of climatic information into forecasting systems may improve the timing and effectiveness of management interventions. Climate-based forecasting models and early-warning systems can help growers identify periods of elevated outbreak risk and reduce unnecessary pesticide applications. Monitoring programs should be linked to economic threshold-based decision-making in order to optimize intervention timing and avoid unnecessary pesticide applications. Such approaches are particularly important in continental and semi-arid fruit-growing regions, where climatic variability may substantially alter pest dynamics among years.

An additional feature distinguishing resilience-oriented management from conventional pest control is the increasing emphasis on adaptive management strategies. Under rapidly changing climatic conditions, static pest management programs based on fixed treatment schedules may become progressively less reliable. Climatic variability alters pest phenology, predator–prey synchronization, overwintering success, and outbreak dynamics, thereby reducing the predictive stability of conventional management thresholds. Adaptive management approaches seek to address this uncertainty through continuous ecological monitoring, flexible decision-making, and iterative adjustment of management strategies according to changing environmental conditions. Rather than relying exclusively on predefined intervention schemes, adaptive systems emphasize learning-oriented management capable of responding dynamically to ecological variability and emerging disturbances.

Holling’s resilience theory has strongly influenced modern adaptive management frameworks by emphasizing the importance of system flexibility, ecological feedbacks, and disturbance buffering in sustainable resource management [77]. In agricultural contexts, adaptive management increasingly involves integration of ecological monitoring, climate forecasting, biodiversity assessment, and risk-based decision support systems. Such approaches may improve the capacity of orchard systems to maintain ecological regulation despite increasing environmental uncertainty.

### 5.2. Climate-Resilient Integrated Mite Management

Resilience-oriented management should be translated into practical strategies specifically targeting phytophagous mite populations in apple orchards. Effective climate-resilient mite management requires integration of selective pesticide use, conservation of predatory mites, adaptive monitoring, and habitat-based regulation.

Selective acaricides should be preferred over broad-spectrum pesticides to minimize disruption of phytoseiid communities and preserve biological regulation capacity. Conservation of predatory mites may be enhanced through reduction in unnecessary pesticide applications, maintenance of suitable overwintering habitats, and preservation of alternative food resources. Habitat diversification strategies should therefore be combined with monitoring-based intervention thresholds and climate-informed decision-making rather than applied as isolated management measures. Such integrated approaches may reduce the probability of secondary mite outbreaks while improving long-term ecological stability under increasing climatic variability.

Notably, resilience-oriented orchard management does not imply complete rejection of chemical interventions. Instead, contemporary ecological frameworks increasingly emphasize reducing ecological disruption while preserving trophic regulation capacity within agroecosystems. Under this perspective, pesticides may still play a role within integrated management systems, but their application should minimize negative effects on beneficial arthropods and long-term ecosystem functionality.

Long-term field studies in Japanese apple orchards showed that conservation of generalist phytoseiid mites significantly suppressed *Tetranychus urticae* populations, supporting the integration of habitat management and biological regulation within climate-resilient IPM programs [78]. These findings demonstrate that resilience-oriented management strategies can provide measurable reductions in phytophagous mite populations when habitat diversification, conservation of predatory mites, and monitoring-based decision-making are implemented simultaneously.

Recent studies suggest that system-oriented regulation represents one of the most promising directions for sustainable orchard protection under climate change [11,79]. System-oriented management considers orchards not as isolated production units, but as dynamic ecological systems embedded within broader landscape and climatic contexts. For apple orchards specifically, resilience-oriented mite management should integrate conservation of *Phytoseiidae* populations, climate-adaptive monitoring of *Panonychus* ulmi and *Tetranychus* spp., selective acaricide use, and habitat-based regulation strategies. These interconnected measures directly address the ecological mechanisms of mite outbreaks discussed in Section 3 and Section 4 and contribute to broader system-oriented management frameworks that enhance agroecosystem resilience. The major ecological components of resilience-oriented orchard management and their functional roles under climatic stress are summarized in Table 2.

Duru et al. emphasized that future agricultural sustainability depends increasingly on systemic redesign of agroecosystems rather than optimization of isolated management components [11]. The authors argued that resilience-oriented agriculture requires integration of ecological complexity, adaptive governance, and multifunctional ecosystem services into production systems. For orchard protection, this means shifting from reactive suppression-oriented management toward ecological frameworks capable of maintaining long-term stability despite climatic stress, resistance evolution, and increasing environmental uncertainty.

Overall, resilience-oriented orchard management represents a conceptual transition from short-term pest suppression toward maintenance of adaptive ecological stability within orchard ecosystems. Ecological intensification, habitat diversification, conservation biological control, climate-adaptive monitoring, and integrated mite management collectively contribute to strengthening trophic resilience and reducing vulnerability of orchard systems under climatic stress.

## 6. Future Perspectives and Research Gaps

Despite the growing number of studies devoted to sustainable orchard protection, important methodological and scientific limitations continue to restrict the development of reliable climate-adaptive mite management strategies [80,81]. One of the principal weaknesses of current research involves the predominance of short-term experimental studies conducted under relatively simplified environmental conditions. Many investigations focus primarily on immediate pest suppression efficiency, whereas long-term ecological responses associated with climatic instability, trophic reorganization, and cumulative environmental stress remain insufficiently understood. As a result, current predictive capacity regarding future dynamics of phytophagous mite outbreaks under climate change remains limited.

Recent analyses increasingly emphasize that long-term ecological datasets are essential for understanding delayed ecosystem responses and non-linear dynamics in agricultural systems. The research [82] demonstrated that ecological processes in agroecosystems frequently exhibit cumulative temporal effects that cannot be adequately detected through short-duration experimental observations. Similar conclusions were reported by Scheffer et al., who emphasized that ecological systems may undergo gradual destabilization before reaching critical transition thresholds that are difficult to predict using simplified short-term analyses [83]. The authors highlighted that climate-driven shifts in species interactions may emerge gradually over multiple years, particularly in perennial agricultural systems characterized by complex trophic relationships and strong seasonal variability. In orchard ecosystems, this limitation is especially important because mite population dynamics are strongly influenced by interactions among climatic conditions, vegetation structure, predator abundance, and agricultural management intensity over extended temporal scales.

Current research is also constrained by the insufficient integration of ecological, climatic, and technological datasets within current orchard protection research. Orchard pest management research often evaluates individual agroecosystem components separately, including pest abundance, pesticide efficacy, climatic trends, or biological control performance [84]. However, sustainable orchard regulation increasingly requires system-level understanding of how these factors interact dynamically under changing environmental conditions. The absence of integrated analytical frameworks substantially limits the ability to predict ecological tipping points and future instability of biological regulation.

Recent advances in digital agriculture and climate-resilient agroecosystem research increasingly emphasize the importance of integrating ecological monitoring, climatic forecasting, and predictive analytical systems within sustainable agricultural management frameworks [85]. Such approaches may substantially improve early-warning capacity for climate-sensitive pest management, particularly in perennial orchard systems exposed to increasing environmental variability.

The fragmented nature of the current literature also remains a major obstacle for the development of resilience-based orchard management frameworks. Many studies continue to evaluate pest management strategies independently from broader landscape and ecosystem processes [86,87]. As a result, ecological interactions among habitat structure, climatic stress, biological regulation, and agricultural intensification are often examined separately rather than as interconnected drivers of agroecosystem stability. This disciplinary fragmentation contributes to inconsistent conclusions regarding the effectiveness of sustainable management strategies across different orchard systems and climatic regions [88].

An additional challenge involves the lack of standardized indicators for evaluating ecological resilience in perennial orchard ecosystems. Although resilience is widely discussed within agroecological research, practical methods for quantifying adaptive ecosystem capacity remain poorly standardized [89]. Recent studies frequently rely on indirect indicators such as pest abundance, biodiversity indices, or short-term productivity metrics, which may not adequately reflect long-term ecological stability under climatic stress conditions [90].

Recent methodological studies increasingly suggest that resilience assessment should incorporate multidimensional indicators capable of simultaneously evaluating climatic sensitivity, trophic stability, biodiversity dynamics, and adaptive ecosystem responses [91]. In particular, monitoring systems based solely on pest population density may fail to detect early destabilization processes occurring within predator–prey interactions or ecosystem functioning. Consequently, future research should prioritize development of integrated resilience metrics suitable for long-term ecological assessment in orchard agroecosystems.

Climate-adaptive monitoring represents another important future direction for sustainable mite management research. Traditional pest surveillance systems are generally based on fixed economic thresholds and historical phenological patterns that may become progressively less reliable under rapidly changing climatic conditions [92]. Rising temperatures, irregular precipitation patterns, and prolonged drought periods increasingly alter developmental timing, overwintering success, and seasonal synchronization among phytophagous mites and their natural enemies [93].

Recent developments in precision agriculture create new opportunities for climate-sensitive ecological monitoring in orchard systems. This study demonstrated that remote sensing technologies combined with machine learning algorithms may substantially improve detection of early plant stress responses associated with pest infestation and climatic disturbance [94]. Similarly, artificial intelligence-assisted ecological forecasting systems may enhance prediction of outbreak risks through integration of climatic, phenological, and environmental datasets [95]. However, implementation of such technologies remains uneven because of high operational costs, limited accessibility, and insufficient availability of long-term ecological calibration data.

Another important research gap concerns the insufficient representation of continental and semi-arid orchard systems within the global literature on sustainable pest management. Most ecological studies on orchard resilience originate from temperate European or North American agricultural systems, whereas climatically vulnerable continental regions remain comparatively underexplored [96]. Nevertheless, increasing thermal stress, irregular precipitation, and chronic water deficits characteristic of semi-arid fruit-growing regions may substantially alter trophic interactions and biological regulation processes compared with humid temperate ecosystems [97].

Recent climatic projections indicate that Central Asian agricultural regions may experience substantial increases in summer temperature extremes and drought frequency in future decades [98]. Such environmental changes may intensify the ecological advantages of phytophagous mites while simultaneously destabilizing populations of predatory arthropods sensitive to microclimatic fluctuations [99]. Despite these risks, region-specific ecological monitoring data for continental orchard systems remain extremely limited. Future research should therefore prioritize long-term field investigations capable of evaluating climate-driven shifts in predator–prey dynamics under semi-arid orchard conditions.

Future research should also increasingly address the role of predictive ecological modeling in sustainable orchard protection. Current pest management strategies frequently remain reactive and intervention-oriented, whereas future climatic instability will likely require anticipatory ecological forecasting approaches capable of identifying ecosystem vulnerability before large-scale pest outbreaks occur. In this context, dynamic simulation models integrating climatic projections, trophic interactions, landscape heterogeneity, and management scenarios may become essential tools for resilience-oriented orchard management.

The major methodological limitations and future research priorities for resilience-oriented orchard management under climate change are summarized in Table 3.

Overall, future progress in sustainable mite management will depend not only on improving individual control measures, but also on strengthening interdisciplinary integration among ecology, climate science, digital agriculture, and long-term ecosystem monitoring. Development of adaptive monitoring systems, integrated resilience indicators, and predictive ecological models may substantially improve the capacity of orchard agroecosystems to withstand climatic instability while maintaining long-term ecological functionality and sustainable fruit production.

## 7. Conclusions

Sustainable management of phytophagous mites in apple orchards increasingly requires a transition from conventional pesticide-dependent protection toward resilience-oriented agroecosystem management. Climatic warming, drought stress, habitat simplification, and agricultural intensification substantially influence trophic interactions and reduce the long-term stability of both chemical and biological control strategies. Under these conditions, mite outbreaks should be considered not only as pest management problems, but also as indicators of broader ecological destabilization within orchard systems.

This review demonstrates that sustainable orchard protection should rely on integrated ecological approaches combining habitat diversification, conservation biological control, adaptive monitoring, ecological intensification, and climate-sensitive management frameworks. Such approaches may improve ecosystem resilience and reduce vulnerability of orchard agroecosystems to climatic variability and resistance development.

At the same time, important scientific and methodological gaps remain unresolved, including insufficient long-term ecological studies, lack of standardized resilience indicators, weak integration of climatic and ecological datasets, and limited research on continental and semi-arid orchard systems. Future progress in sustainable mite management will therefore depend on interdisciplinary and system-oriented research capable of integrating ecological monitoring, predictive modeling, and adaptive management into comprehensive climate-resilient orchard protection strategies.

## Figures and Tables

**Table 1:** Ecological limitations of conventional and biological mite management strategies in orchard systems.

Management Approach	Advantages	Ecological Limitations	Long-Term Risks
Conventional chemical control	Rapid pest suppression; operational simplicity	Non-target toxicity; trophic disruption; resistance pressure	Secondary outbreaks; ecological destabilization
Biological control	Reduced pesticide dependence; ecological compatibility	Climate sensitivity; unstable predator establishment	Inconsistent regulation under climatic stress
Conservation biological control	Supports biodiversity and trophic buffering	Delayed effectiveness; habitat dependence	Variable performance among orchard systems
Integrated management approaches	Improved resilience and reduced chemical pressure	Higher management complexity; monitoring requirements	Economic and operational constraints

**Table 2:** Components of resilience-oriented orchard management under climatic stress.

Ecological Component	Functional Role in Orchard Systems	Contribution to Resilience	Main Limitations
Ecological intensification	Strengthening ecosystem services and trophic regulation	Reduced ecological disturbance; improved adaptive capacity	Requires long-term system redesign
Habitat diversification	Increasing habitat heterogeneity and refugia for natural enemies	Improved biodiversity and trophic buffering	Context-dependent effectiveness
Conservation biological control	Preservation of naturally occurring beneficial arthropods	Stabilization of predator–prey interactions	Delayed and variable response
Adaptive management	Flexible adjustment of management practices under climatic variability	Increased responsiveness to environmental change	Requires continuous monitoring and expertise
System-oriented regulation	Integration of ecological, climatic, and management factors	Long-term agroecosystem resilience	High operational complexity
Climate-adaptive monitoring	Early detection of mite outbreaks and support of management decisions	Improved intervention timing and reduced pesticide use	Requires monitoring infrastructure and technical expertise
Climate-resilient integrated mite management	Integration of monitoring, selective acaricides, biological control, and habitat management	Improved long-term mite suppression and trophic stability	Requires coordinated implementation and ecological knowledge

**Table 3:** Major research gaps and future priorities for resilience-oriented orchard management under climate change.

Research Gap	Current Limitation	Future Research Direction
Lack of long-term ecosystem datasets	Predominance of short-term experimental studies	Multi-year ecological monitoring in perennial orchard systems
Fragmented interdisciplinary research	Weak integration among ecology, climatology, and pest management	Development of system-oriented analytical frameworks
Absence of standardized resilience indicators	Limited methodologies for evaluating adaptive ecosystem stability	Creation of integrated ecological resilience metrics
Insufficient climate-adaptive monitoring	Traditional thresholds poorly reflect climatic variability	AI-assisted monitoring and predictive ecological forecasting
Limited regional representation	Underrepresentation of continental and semi-arid orchard systems	Region-specific resilience studies under climate stress
Weak integration of digital technologies	Limited use of remote sensing and ecological modeling	Precision agriculture and ecosystem-based forecasting systems

## Data Availability

No new data were created or analyzed in this study.
